# Simulation Study of Canal Switching in BPPV

**DOI:** 10.3389/fneur.2022.944703

**Published:** 2022-07-13

**Authors:** Shuzhi Wu, Jianxin Li, Mi Zhou, Xiaokai Yang

**Affiliations:** ^1^Neurology Department, Third Affiliated Hospital of Shanghai University, Wenzhou Third Clinical Institute Affiliated to Wenzhou Medical University, Wenzhou People's Hospital, Wenzhou, China; ^2^Third Affiliated Hospital of Shanghai University, Wenzhou Third Clinical Institute Affiliated to Wenzhou Medical University, Wenzhou People's Hospital, Wenzhou, China

**Keywords:** BPPV, otolith, virtual simulation, therapy, Epley maneuver, canal switching

## Abstract

The objective of this research was to investigate the mechanism of canal switching in benign paroxysmal positional vertigo through a virtual simulation model. Using Unity 3D software and a built-in NVIDIA Physx physics engine, the virtual simulation software is developed using a browser-server architecture, and different models are imported. Based on the benign paroxysmal positional vertigo virtual simulation model, we constructed five different virtual reality scenes of diagnosis and treatment, set otoliths in different positions of the semicircular canals, and analyzed the effects of diagnostic and therapeutic procedures on otolith location. Through the analysis of otolith movement in five virtual scenes, we found that canal switching may be caused by otoliths in the utricle entering the semicircular canal in the supine position. Then, we used different methods to reposition the otolith, improved the repositioning maneuver, and explored in depth the mechanism of the canal switching. The results showed that the main reason for the canal switch is that in the supine position, the otolith in the utricle enters the semicircular canal. The repositioning maneuvers, including the Epley maneuver and Barbecue maneuver, will not directly lead to the canal switch in the ipsilateral inner ear. The supine roll maneuver leads to the otolith in the utricle entering the posterior or lateral semicircular canal, which is the most likely mechanism for canal switching.

## 1. Introduction

Benign paroxysmal positional vertigo (BPPV) is the most common disease that causes peripheral vertigo clinically. BPPV of the posterior semicircular canal is the most common, followed by BPPV of the lateral semicircular canal, while BPPV of the superior semicircular canal is rare. The pathogenesis of BPPV is free-floating otolith deposits in the semicircular canal or adhered to the cupula. As the head position changes, the otolith settles under the action of gravity, which directly or indirectly acts on the crista ampullaris, thus inducing a vertigo attack. The preferred treatment for BPPV is to induce vertigo and nystagmus by changing the head position to determine the location of the otolith and then returning the displaced otolith to the utricle through a series of changes in the head position. In the process of diagnosis and treatment, the phenomenon of otolith shifting from one semicircular canal to another is called canal switching (1). The shift of otolith from the long arm side to the short arm side or from the short arm side to the long arm side of the same semicircular canal shall also be regarded as canal switching. The phenomenon in which the otoliths reposition to the utricle re-enter the same semicircular canal is called otolith reentry. The occurrence of canal switching complicates the diagnosis and treatment of BPPV, which can easily lead to misdiagnosis and missed diagnosis and is one of the important reasons for unsuccessful repositioning treatment. Currently, there is no effective method to prevent the occurrence of canal switching. Canal switching has been reported to occur in posterior, anterior, and lateral semicircular canal BPPV with an incidence of 2.3–16% (2–4). At present, the most common canal conversion is between the posterior and lateral semicircular canals, followed by canal conversion between the posterior and anterior semicircular canals, while canal conversion between the anterior and lateral semicircular canals has not been reported (3). In recent years, increasing attention has been given to the study of canal switching in BPPV, but the mechanism is not clear. At present, it is generally believed that the switch of the otolith semicircular canal is caused by the repositioning treatment process. Some scholars believe that the irregularity of the repositioning technique is the main reason for the canal switching. However, according to research on the swivel chair system, canal switching will occur even in a standardized treatment. The factors affecting the canal switching are not completely clear, which may be related to the anatomical structure of the semicircular canal, the repositioning method, and the time of reexamining the diagnostic maneuver. It is believed that the final step of the Epley maneuver is to sit upright, and at this point, the opening of the common crus is higher than that of the single crus. Therefore, under the action of gravity, otolith particles can easily enter the lateral semicircular canal and lead to BPPV of the lateral semicircular canal. In addition, for the Barbecue maneuver, when lying on the healthy side, the opening of the single crus on the affected side is higher than the opening of the common crus, and under the action of gravity, otolith particles can easily enter the common crus. However, these explanations can be found to be unreasonable by the observation of the spatial posture of the semicircular canal in the three-dimensional spatial coordinate system (5). Although the common crus is very close to the lateral semicircular canal at the opening of the utricle, regardless of whether it is the upright position in the last step of the Epley maneuver or the lateral position in the Barbecue maneuver, the otolith will not migrate from one semicircular canal to another. There is a lack of adequate understanding of how otoliths in the utricle enter the semicircular canal during diagnosis and treatment. The main reason for this is the difficulty in visualizing and determining the movement and position of the otolith. With the application of virtual simulation technology in medicine, the BPPV virtual simulation model can observe otolith movement induced by diagnostic maneuvers and therapy maneuvers, and it has become one of the important tools to study BPPV (6). A study based on the BPPV virtual simulation model found that the otoliths located in the utricle in the supine position would enter the semicircular canal (7), and it was hypothesized that this could be the key reason for the canal switching. In this article, based on the BPPV virtual simulation model, the otoliths were set in different positions of the semicircular canals to analyze the changes in otolith position during the diagnosis and treatment of BPPV and to explore the mechanism of canal switching.

## 2. Materials and Methods

### 2.1. Establishment of a BPPV Virtual Simulation Model

The BPPV virtual simulation model is based on Unity 3D software (version 2020.3) and a built-in NVIDIA Physx physical engine developed using a browser and server architecture. The software supports importing models, rotational representation of Euler angles and quaternions, and the coordinate system can be the world coordinate system or object positioning. The ratio of the virtual semicircular canal to the real semicircular canal is 1:1. Before the operation, we imported the membrane semicircular canal model (7), which had been built in the early stage, and the standard spatial coordinate system was set to the X, Y, and Z axes. The virtual semicircular canal model can be adjusted for many parameters, such as resistance, friction coefficient, and buoyancy. In the model, different types of BPPV can be constructed by clicking the mouse and adding otoliths at any of the specified locations.

### 2.2. Parameter Settings

The radius of otoliths ranges from 0.5 to 15 nm, with an average radius of 7.5 nm. The density of otolith is 2.71 *g*/*cm*^3^, the density of the endolymphatic fluid is 1 *g*/*cm*^3^, and the buoyancy is set at 3.62 *m*/*s*^2^ (8–10). The resistance parameters and friction coefficient were adjusted to make the otolith settlement speed close to 0.2 *mm*/*s* (11, 12).

### 2.3. Otolith Position Setting

In the upright position, otoliths are set in different semicircular canals, including posterior, lateral, and superior semicircular canals, the short arm side and long arm side, as well as in different positions of the utricle, and naturally, settle under the action of gravity. For cupulolithiasis, refer to canalolithiasis with otolith in the ampulla.

### 2.4. Analysis of Otolith Repositioning Treatment

The otoliths were set in different positions to observe and analyze the trajectory of the otoliths during the Epley maneuver and the Barbecue maneuver.

### 2.5. Analysis of the Head Position of the Otolith in the Utricle Into the Semicircular Canal

The etroflexion angle and its effect on the entry of the otolith located in the utricle into the semicircular canal were analyzed.

## 3. Results

### 3.1. The Effect of the Right Epley Maneuver

**Figure 1:**
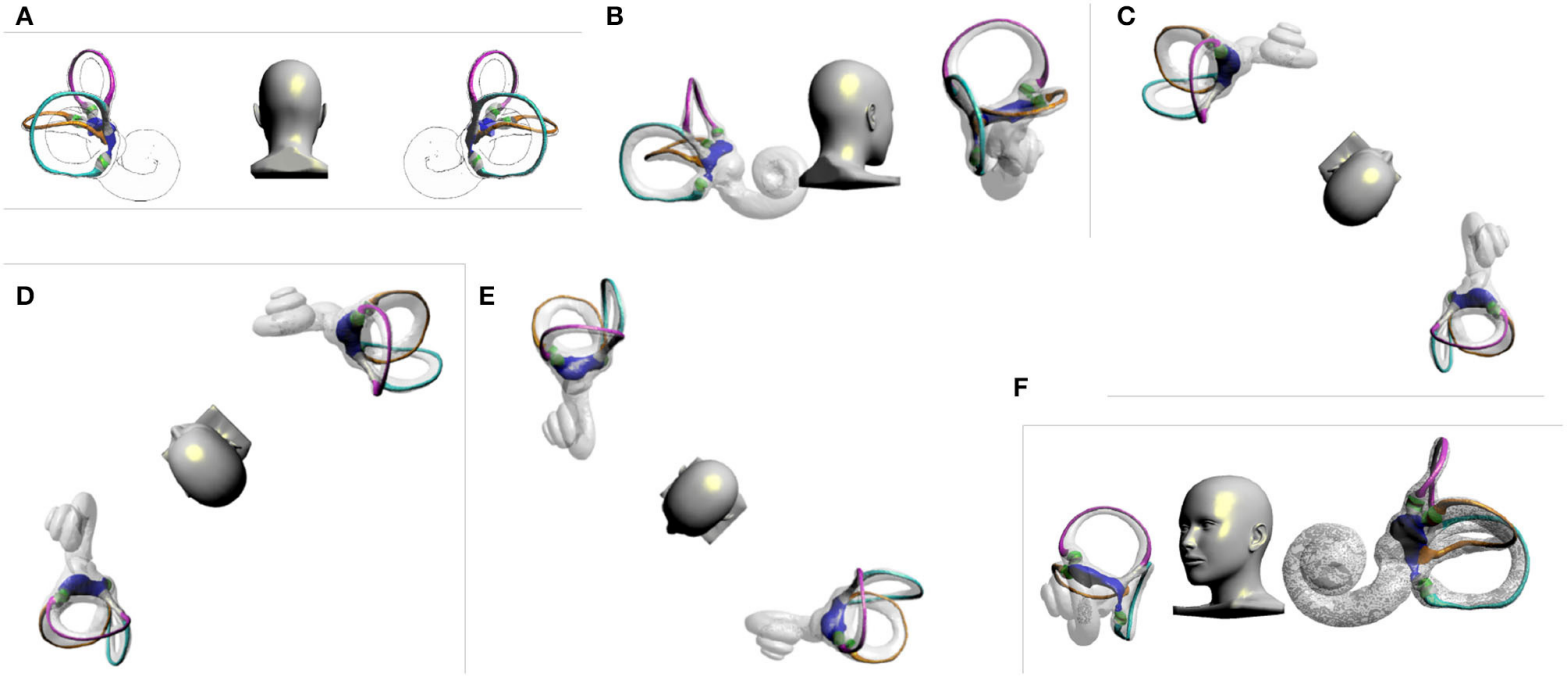
Right Epley maneuver. **(A)** Upright position. **(B)** Head turned 45 to the right. **(C)** The patient quickly lies back, supine, with his head hanging at 30°. **(D)** Head turns 90° to the left. **(E)** Head continues to turn 90° to the left. **(F)** Return to sitting position.

Euler angles are defined as continuous plane rotation angles about the x, y, and z axes, while quaternion is another spatial rotation representation. A quaternion number is represented in the form *a* + *bi* + *cj* + *dk*, where Parts a, b, c, and d are real numbers, and Parts i, j, and k are the basis elements. The head position of the Epley maneuver is quite special and difficult to describe by Euler angles, and therefore, it is necessary to move the head position by calculating the quaternions. Taking the right Epley maneuver as an example (Figure 1), the quaternions corresponding to steps B-F are 0.924 − 0.383*k*, 0.462 + 0.8*i* + 0.331*j* − 0.191*k*, 0.462 + 0.8*i* − 0.331*j* + 0.191*k*, 0.271 + 0.271*i* − 0.653*j* + 0.653*k*, respectively. The otolith in the short arm of the right lateral semicircular canal will enter the utricle and shift to the right superior semicircular canal in step E, and the otolith will re-enter the utricle when sitting up. The otoliths in the long arm side of the right lateral and posterior semicircular canals enter the utricle in step E. The otolith in the long arm side of the right semicircular canal enters the utricle in step F. The otolith in the right utricle enters the right superior semicircular canal in step D, and the otolith re-enters the utricle when the sitting position is restored. Based on the virtual simulation model to observe the effect of the right Epley maneuver on the position of the otoliths, we found that the otoliths were located in the right inner ear, and only the otoliths in the short arm of the right posterior semicircular canal could not return to the utricle. The otoliths located in the short arm of the left lateral semicircular canal and the left utricle enter the long arm side of the left superior semicircular canal in step C and stay in it. The Epley maneuver does not lead the otoliths in other positions of the left semicircular canal to return to the utricle (Table 1). Generally, the right Epley maneuver repositioning operation returns the otoliths located in the long-arm side of the posterior semicircular canal, the long-arm side of the right superior semicircular canal, and the short-arm side and long-arm side of the right lateral semicircular canal to the utricle. However, it also has shortcomings and defects, which may lead to otoliths in the short arm of the left lateral semicircular canal and the left utricle entering the superior semicircular canal, resulting in BPPV of the left superior semicircular canal (**Figure 3A**), and it is impossible to reposition the otolith in the short arm of the posterior semicircular canal. Animations of the otolith movement are provided in the Appendix.

**Table 1:** Changes of otolith position at different positions during the right Epley maneuver.

**Step A**	**Step B**	**Step C**	**Step D**	**Step E**	**Step F**
RPSa	RPSa	RPSa	RPSa	RPSa	RPSa
RPLa	RPLa	RPLa	RPLa	RU	RU
RALa	RALa	RALa	RALa	RU	RU
RHSa	RHSa	RALa	RALa	RALa	RU
RHLa	RHLa	RHLa	RHLa	RU	RU
LHSa	LHSa	LALa	LALa	LALa	LALa
LU	LU	LALa	LALa	LALa	LALa
RU	RU	RU	RALa	RALa	RU

*The naming rules are left- and right-side anatomical structure-specific positions. L, left side; R, right side; P, posterior semicircular canal; H, horizontal semicircular canal; A, anterior semicircular canal; Sa, short arm side; La, long arm side; U, utricle*.

### 3.2. The Effect of the Right Barbecue Maneuver

**Figure 2:**
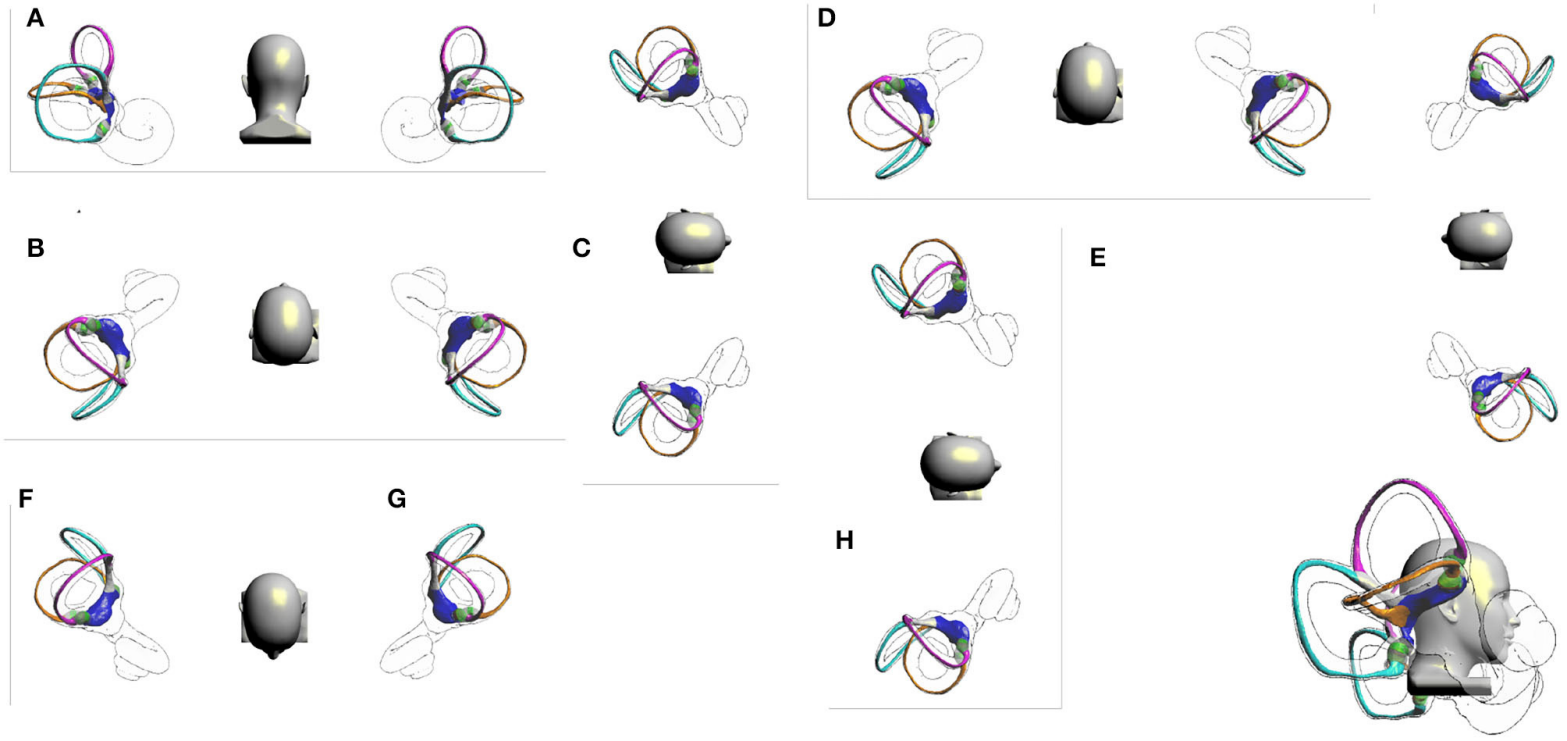
Right Barbecue maneuver. **(A)** Upright position. **(B)** Rapidly laid back to the supine position. **(C)** Turn over 90° to the right, right lateral position. **(D)** Turn over 90° to the left, supine position. **(E)** Continue turning over 90°, left lateral position. **(F)** Continue turning over 90°, prone position. **(G)** Continue turning over 90°, left lateral position. **(H)** Sit up sideways and return to an upright position.

**Figure 3:**
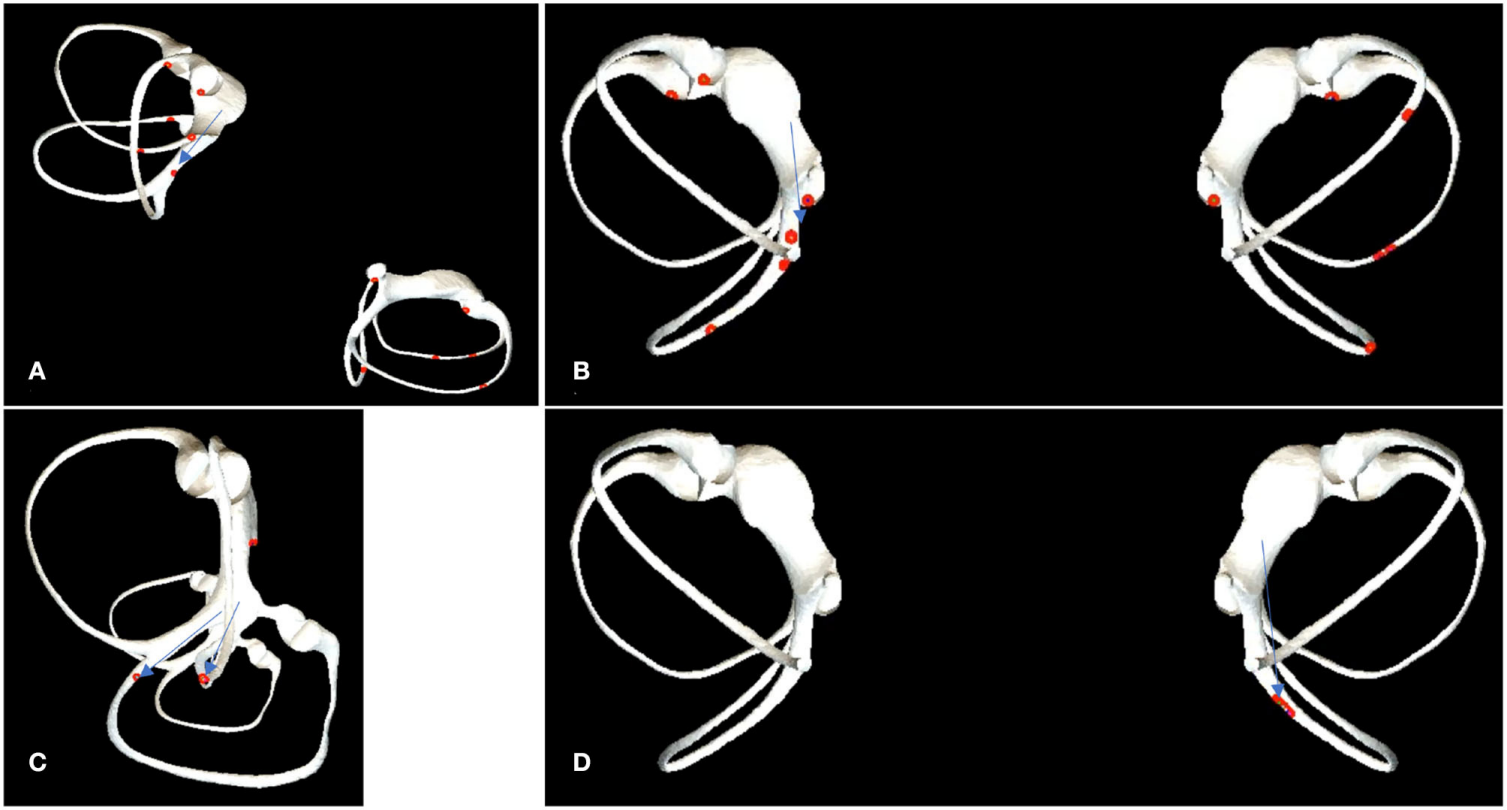
Canal switch scenarios. **(A)** The right Epley maneuver, step C. The patient quickly lies back, supine, with his head hanging at 30°. The otoliths in the short arm of the left lateral semicircular canal and the left utricle enter the superior semicircular canal, resulting in BPPV of the left superior semicircular canal. **(B)** In the right Barbecue maneuver, step D. Supine position, the otoliths in the short arm of the left lateral semicircular canal and the left utricle enter the posterior semicircular canal, resulting in BPPV of the left posterior semicircular canal. **(C)** Head tilted back 70°, the otoliths in the utricle enter the posterior or the lateral semicircular canal. **(D)** Head tilted back 90°, the otoliths in the utricle enter the posterior semicircular canal.

Take the left Barbecue maneuver as an example (Figure 2). In Step D, the otolith in the right superior semicircular canal moves away from the ampulla. In Step E, the otolith in the long arm side and short arm side of the right lateral semicircular canal enters the utricle, and the otolith in the right posterior semicircular canal enters the superior semicircular canal near the common crus. In the supine position, the otolith in the utricle may enter the posterior semicircular canal through the common crus. In the supine position, the otolith in the posterior semicircular canal moves away from the ampulla, and its moving distance is longer. Because the posterior semicircular canal is not parallel to the gravity direction while in the supine position, the otolith moves slowly, and it needs to be kept for more than 45 s. Generally, the right Barbecue maneuver can be used to reposition the otoliths in the following locations, including the long-arm and short-arm side of the right lateral semicircular canal, the long-arm side of the right posterior semicircular canal, the right superior semicircular canal, and the short-arm side and long-arm side of the left lateral semicircular canal. However, it also has shortcomings and defects, which may lead to otoliths in the short arm of the left lateral semicircular canal and the left utricle entering the posterior semicircular canal, resulting in BPPV of the left posterior semicircular canal (Figure 3B). The right Barbecue maneuver cannot reposition the otoliths in the short arm of the posterior semicircular canal (Table 2). Animations of the otolith movement are provided in the Appendix.

**Table 2:** Changes of otolith position at different positions during the right barbecue maneuver.

**Step A**	**Step B**	**Step C**	**Step D**	**Step E**	**Step F**	**Step G**	**Step H**
RPSa	RPSa	RPSa	RPSa	RPSa	RPSa	RPSa	RPSa
RPLa	RPLa	RPLa	RPLa	RALa	RU	RHSa	RHSa
RALa	RALa	RALa	RALa	RALa	RALa	RALa	RU
RHSa	RHSa	RHSa	RHSa	RU	RU	RHSa	RHSa
RHLa	RHLa	RHLa	RHLa	RU	RU	RHSa	RHSa
RU	RPLa	RPLa	RPLa	RALa	RU	RHSa	RHSa
LHSa	LHSa	LU	LPLa	LPLa	LPLa	LPLa	LPLa
LHLa	LHLa	LU	LPLa	LPLa	LPLa	LPLa	LPLa
LHLa*	LHLa	LHLa	LHLa	LHLa	LHLa	LHLa	LHLa
LU	LU	LU	LPLa	LPLa	LPLa	LPLa	LPLa
LU	LPLa	LALa	LALa	LPLa	LPLa	LPLa	LPLa
LPLa	LPLa	LALa	LALa	LPLa	LPLa	LPLa	LPLa
LPSa	LPSa	LPSa	LPSa	LPSa	LPSa	LPSa	LPSa

The naming rules are left- and right-side anatomical structure-specific positions. L, left side; R, right side; P, posterior semicircular canal; H, horizontal semicircular canal; A, anterior semicircular canal; Sa, short arm side; La, long arm side; U, utricle. LHLa* is the otolith located in the ampulla of the long arm of the left lateral semicircular canal.

### 3.3. The Head Position of Otoliths in the Utricle Entering the Semicircular Canal

The common crus, the long arm of the lateral semicircular canal, and the short arm of the posterior semicircular canal are adjacent to each other at their utricle openings. In the upright position, with the utricle as a reference, the common crus is in a high position, the lateral semicircular canal is at the same level, and the short arm of the posterior semicircular canal is in a low position. When the position of the head changes, the position of the free-floating otolith also changes. In the upright position, the short arm of the posterior semicircular canal lies beneath the utricle, where the otolith is most easily accessible. When the head is tilted back at an angle of more than 25°, the otolith may enter the lateral semicircular canal. When the head is tilted back at an angle of more than 60°, the common crus begins to lie below the utricle; however, because the otolith is located at the bottom of the utricle, it is still difficult to enter the semicircular canal at this time, but the lateral semicircular canal. When the head is tilted back at an angle of more than 70° but less than 90°, the otolith will enter the posterior or the lateral semicircular canal (Figure 3C). When the head is tilted back at an angle of more than 90°, the otolith will enter the posterior semicircular canal *via* the common crus but no longer enter the lateral semicircular canal (Figure 3D). When the head is tilted back at an angle of more than 130°, a portion of the otolith will begin to pass through the common crus into the superior semicircular canal.

## 4. Discussion

The following five virtual scenes are helpful for studying the mechanism of canal switching. 1. Tilt back will make the otoliths in the utricle enter the semicircular canal. The otolith enters different semicircular canals when tilted backward at different angles. With the head elevated 30°–35° while lying supine, the otolith will enter the posterior semicircular canal through the common crus. In the supine position, depending on the position of the otolith in the utricle, it may enter the posterior or the lateral semicircular canal. After repositioning treatment of lateral semicircular canal BPPV, the supine roll maneuver is usually re-examined, which may induce otoliths that have been repositioned to the utricle to enter the posterior semicircular canal, thus causing canal conversion. If the supine roll maneuver is used first, followed by the Dix-Hallpike maneuver, the canal switching may be found immediately. In contrast, if the Dix-Hallpike maneuver is used first and then the supine roll maneuver is used, the canal switching cannot be found immediately. 2. In the Dix-Hallpike maneuver, the otolith in the opposite utricle will enter the superior semicircular canal through the common crus, but upon return to the sitting position, the otolith returns to the utricle and does not cause a canal switching. It has been reported that the Dix-Hallpike maneuver immediately after repositioning is related to canal switching. We think this may be just a superficial phenomenon, and it is more likely that the supine roll maneuver reexamination is the main reason. The Dix-Hallpike maneuver can be reexamined immediately after repositioning. 3. During the treatment of the Epley maneuver, otoliths in the contralateral utricle will enter the superior semicircular canal through the common crus, which will induce BPPV of the superior semicircular canal. However, ipsilateral canal switching will not occur. For multicanal canalithiasis, especially those with bilateral semicircular canal otoliths, after treatment of one side, it is necessary to bow the head for at least 15 min to allow the otolith to adhere to the macula of the utricle again before treatment of the other side. 4. The Barbecue maneuver will make the otolith in the contralateral utricle enter the posterior semicircular canal. Step G will make the otoliths in the utricle enter the short arm of the ipsilateral lateral semicircular canal, which is unnecessary. 5. In the upright position, the bottom of the utricle is nearly parallel to the lateral semicircular canal, and the posterior part of the utricle and the short arm of the posterior semicircular canal are in a low position, where otoliths are easily deposited. To prevent otoliths in the utricle from entering the short arm side of the posterior semicircular canal and adhering to the macula of the utricle, it is important to bow more than 30° when returning to the sitting position.

**Figure 4:**
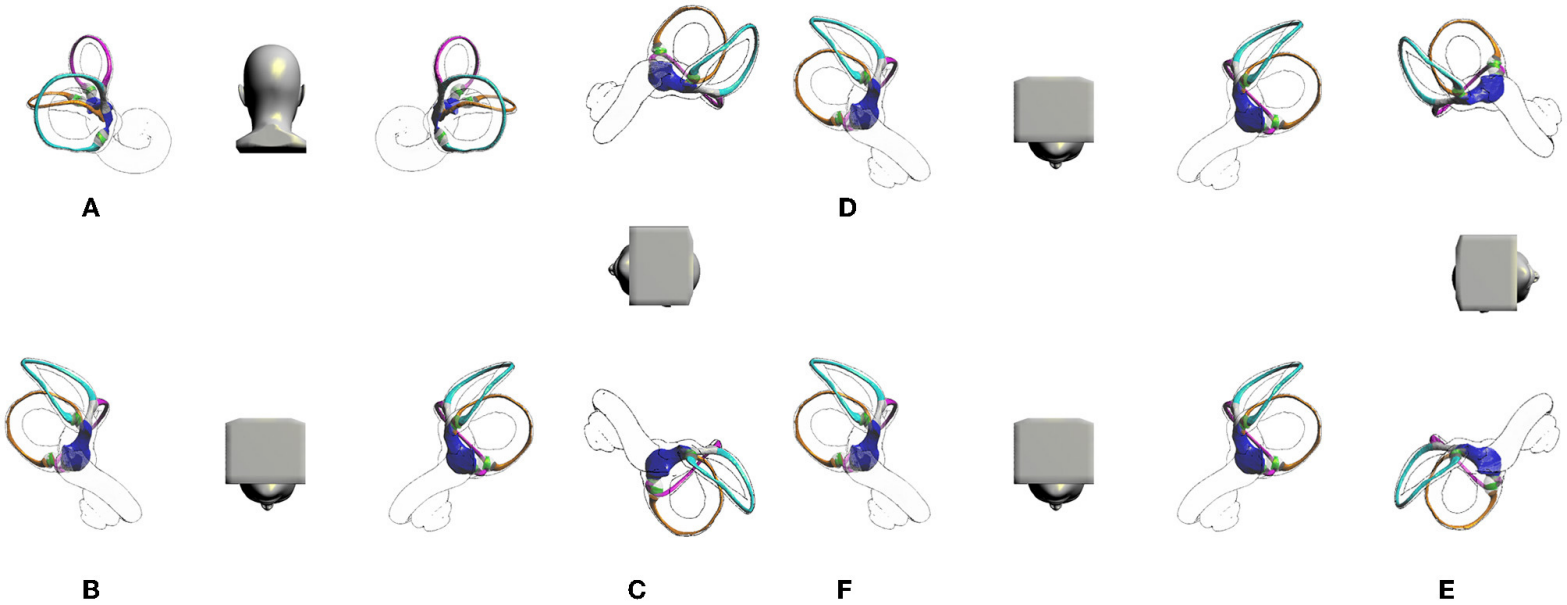
Prone roll maneuver. **(A)** Upright position. **(B)** Tilted forward 90°, prone position. **(C)** Turn head 90° to one side (right lateral position). **(D)** Return to the prone position. **(E)** Turn head 90° to the other side (left lateral position). **(F)** Return to the prone position.

Neither the Epley maneuver nor the Barbecue maneuver will lead to the canal switching in the ipsilateral inner ear. Diagnosis with the Dix-Hallpike maneuver is also safe. In contrast, the supine roll maneuver is the most likely cause of canal switching between the posterior and lateral semicircular canal and otolith reentry. The relatively high probability of migration of otoliths from the posterior semicircular canal to the ipsilateral lateral semicircular canal may be because BPPV usually occurs in the posterior semicircular canal. After treatment, the supine roll maneuver needs to be performed again. If the head position is not fully raised, the otolith may re-enter semicircular canals. Otoliths in the lateral semicircular canal also tend to migrate to the posterior semicircular canal but are less common, mainly because of the lower incidence of BPPV in the lateral semicircular canal. The lateral semicircular canal also easily migrates to the posterior semicircular canal. The main reason may be the low incidence of BPPV in the external semicircular canallateral semicircular canal. The canal switching between the ipsilateral posterior semicircular canal and the superior semicircular canal is difficult to explain. In fact, superior semicircular canal BPPV is so rare that its affected side is not easy to determine and can easily be missed and misdiagnosed. In addition, when the otolith in the posterior semicircular canal flows to the ampulla, which produces inhibitory stimulation, it is often misdiagnosed as BPPV of the superior semicircular canal. A reasonable explanation for these phenomena is that the Epley maneuver can induce otoliths in the short arm of the contralateral lateral semicircular canal and contralateral utricle to enter the superior semicircular canal. How to prevent canal switching, including otolith reentry, is an important topic to be addressed in the current diagnosis and treatment of BPPV. The key is to improve the supine roll maneuver to avoid the head leaning back. In the process of treatment, the Bow and Lean Test (BLT) can be used instead of the supine roll maneuver (13, 14). The BLT is performed as follows: bend your head for 90° first, and then turn your head up for 45° (13) or 60° (15). When the head is tilted forward at 90°, the otolith of the lateral semicircular canal semicircular canal moves to the ampulla, which is an exciting stimulus and can induce horizontal nystagmus. The direction of nystagmus refers to the affected side. The movement of the otolith in the posterior semicircular canal away from the ampulla is an inhibitory stimulus, that can induce horizontal nystagmus. The direction of nystagmus refers to the healthy side. The opposite is true in the case of lateral semicircular canal cupulolithiasis. The BLT only needs to observe the direction of the nystagmus to make the localization diagnosis, while the supine roll maneuver also needs to compare the nystagmus intensity between the left and right sides. Clinically, it is generally considered that the former has higher positioning accuracy (15). However, the BLT requires a supine roll maneuver to judge whether it is cupulolithiasis. or canalithiasis, which is a supplement to the supine roll maneuver, but it cannot be used as an independent diagnostic test for BPPV of the lateral semicircular canal. Moreover, the BPPV virtual simulation test shows that the otolith in the lateral semicircular canal enters the ampulla when the head is tilted forward at 90°, and the otolith located in the ampulla does not easily come out when leaning back. Use the prone roll maneuver, tilt the head forward 90°, turn 90° to one side, and return to the prone position, turn the head 90° to the other side, and return to the prone position (Figure 4). Although the BLT suggests that the head-down time should be kept for at least 2 min (16), the results of the BPPV virtual simulation model show that it takes less than 30 s for the otolith to move to the ampulla and reach the ampulla, so we think that it is enough to bend the head for 30 s, or keep the head-down time until the dizziness nystagmus disappears. Similarly, the maintenance time of other steps is also 30 s. Turning the head from side to side in the prone position, the otoliths in the semicircular canal located on the lower ear side moved away from the ampulla as an inhibitory stimulus with apogeotropic horizontal nystagmus; the otoliths in the semicircular canal located on the upper ear side were located at the base of the crista ampullaris and produced no stimulation. The otoliths adhered to the cupula on the side of the upper ear moved toward the ampulla as an excitatory stimulus with continuous apogeotropic horizontal nystagmus; the otoliths adhered to the cupula on the side of the lower ear moved away from the ampula as an inhibitory stimulus with continuous apogeotropic horizontal nystagmus. Therefore, the presence of bilateral apogeotropic horizontal nystagmus or persistent nystagmus can be judged as cupulolithiasis. The zero plane (16) helps to further clarify cupulolithiasis. Therefore, prone nystagmus combined with left and right head-turn nystagmus can achieve the goal of otolith localization and diagnosis, thus replacing the supine roll maneuver.

The main reason for the canal switch is that the otolith in the utricle in the supine position enters the semicircular canal. The repositioning methods include the Epley maneuver and the Barbecue maneuver, neither of which will directly lead to ipsilateral canal switching. However, the supine position of the supine roll maneuver easily leads to the otolith located in the utricle entering the posterior semicircular canal or the lateral semicircular canal, which may be the most likely mechanism of canal switch. The prone roll maneuver instead of the supine roll maneuver can effectively prevent canal switching. At the same time, the diagnostic test can be reexamined immediately after repositioning, which will not only decrease the incidence rate of otolith reentry and canal switching but also improve the efficiency of diagnosis and treatment.

## Data Availability Statement

The original contributions presented in the study are included in the article/Supplementary Material, further inquiries can be directed to the corresponding author.

## Ethics Statement

The studies involving human participants were reviewed and approved by the Ethics Committee of Wenzhou People's Hospital. Written informed consent for participation was not required for this study in accordance with the national legislation and the institutional requirements.

## Author Contributions

XY conceived designed the experiment. XY, SW, JL, and MZ wrote the manuscript. XY and SW conducted the experiment. All the authors read and approved the manuscript.

## Funding

This study was funded by the foundation of the Wenzhou Science and Technology Bureau (Grant No. Y2020420) to SW, the Natural Science Foundation of Zhejiang Province (Grant No. LSY19H090002) to XY, and the Higher Education Teaching Reform Project of Wenzhou Medical University (Grant No. JG2020136) to XY.

## Conflict of Interest

The authors declare that the research was conducted in the absence of any commercial or financial relationships that could be construed as a potential conflict of interest.

## Publisher's Note

All claims expressed in this article are solely those of the authors and do not necessarily represent those of their affiliated organizations, or those of the publisher, the editors and the reviewers. Any product that may be evaluated in this article, or claim that may be made by its manufacturer, is not guaranteed or endorsed by the publisher.
