# Supplementary material for: Simulation Study of Canal Switching in BPPV
Source: Front Neurol. 2022 Jul 13;13:944703. doi: 10.3389/fneur.2022.944703 (PMC9326062; doi:10.3389/fneur.2022.944703)
Supplement: Supplementary file 1 [file Presentation_1.PPTX]

## Slide 1
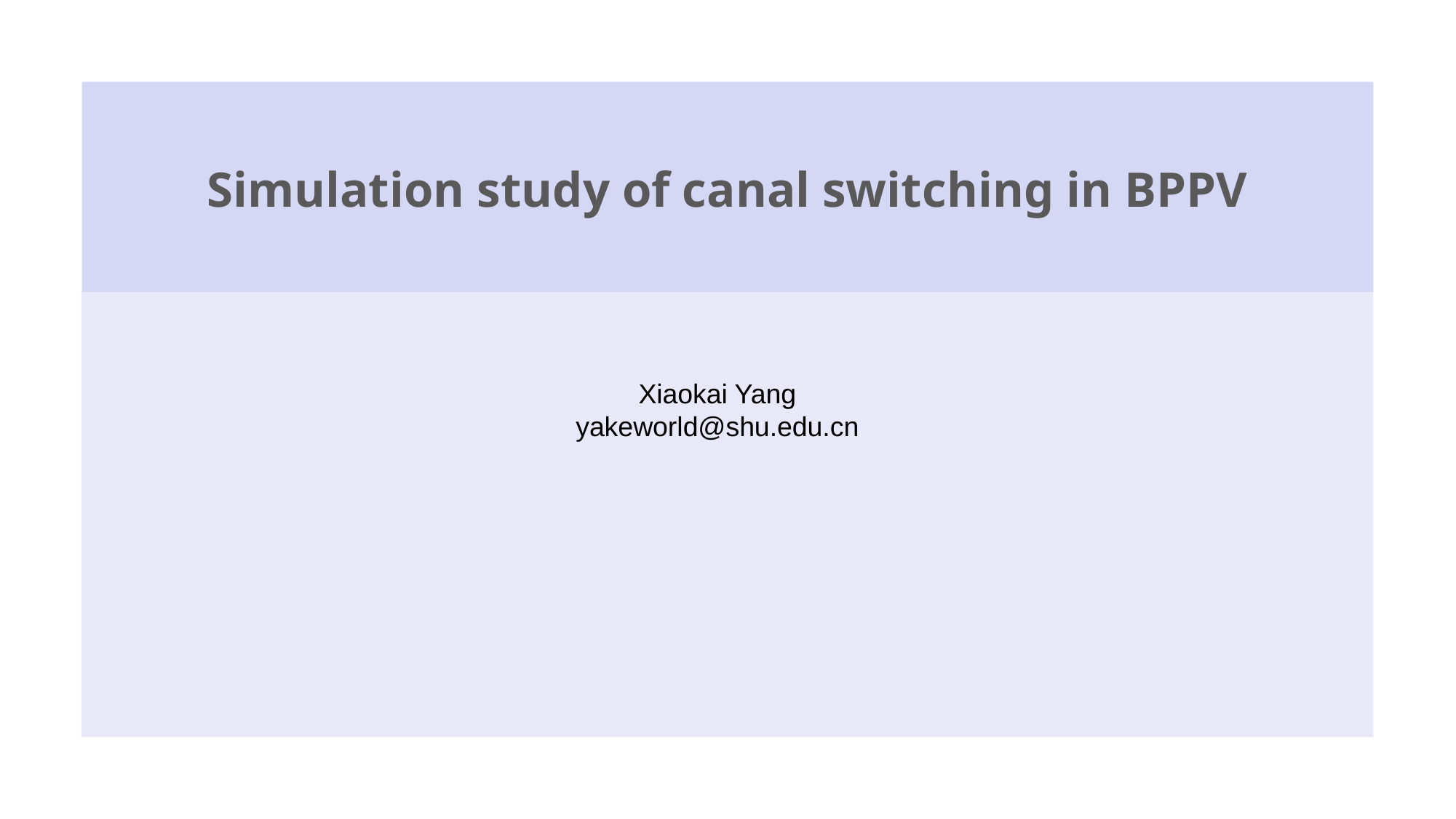

# Simulation study of canal switching in BPPV
Xiaokai Yang
yakeworld@shu.edu.cn

## Slide 2
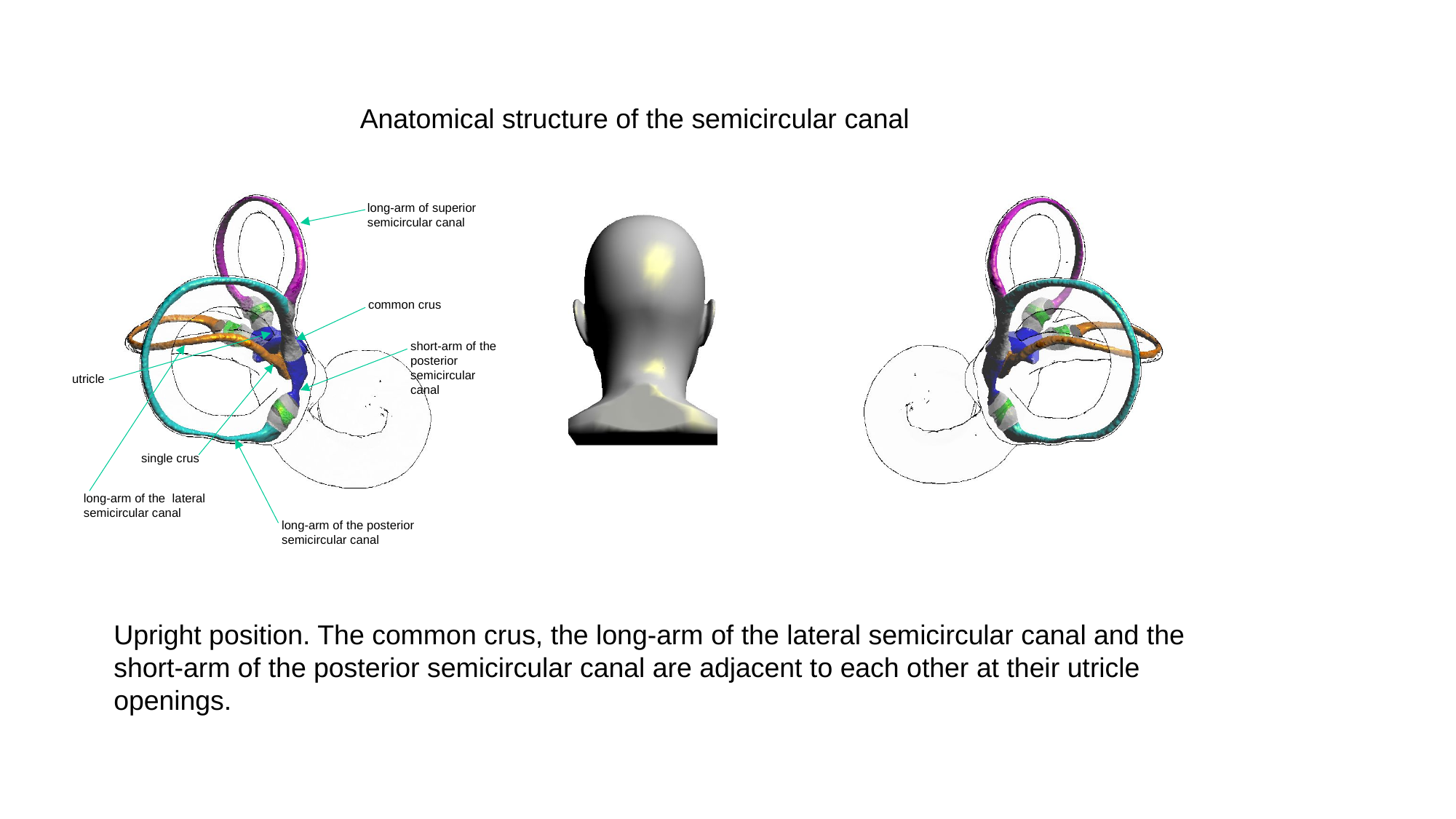

Anatomical structure of the semicircular canal
long-arm of superior semicircular canal
common crus
short-arm of the posterior semicircular canal
utricle
single crus
long-arm of the lateral semicircular canal
long-arm of the posterior semicircular canal
Upright position. The common crus, the long-arm of the lateral semicircular canal and the short-arm of the posterior semicircular canal are adjacent to each other at their utricle openings.

## Slide 3
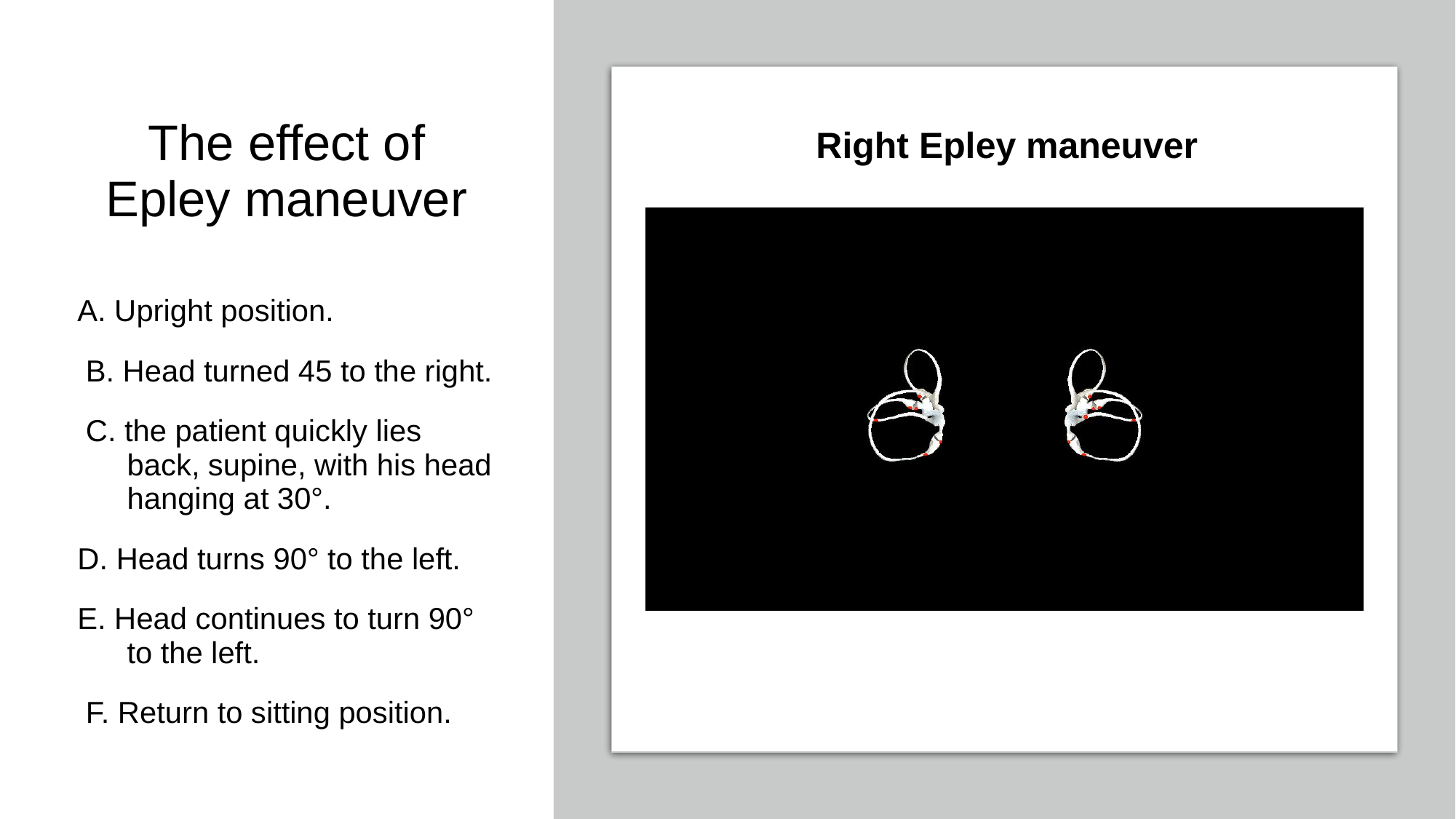

# The effect of Epley maneuver
Right Epley maneuver
A. Upright position.
 B. Head turned 45 to the right.
 C. the patient quickly lies back, supine, with his head hanging at 30°.
D. Head turns 90° to the left.
E. Head continues to turn 90° to the left.
 F. Return to sitting position.

## Slide 4
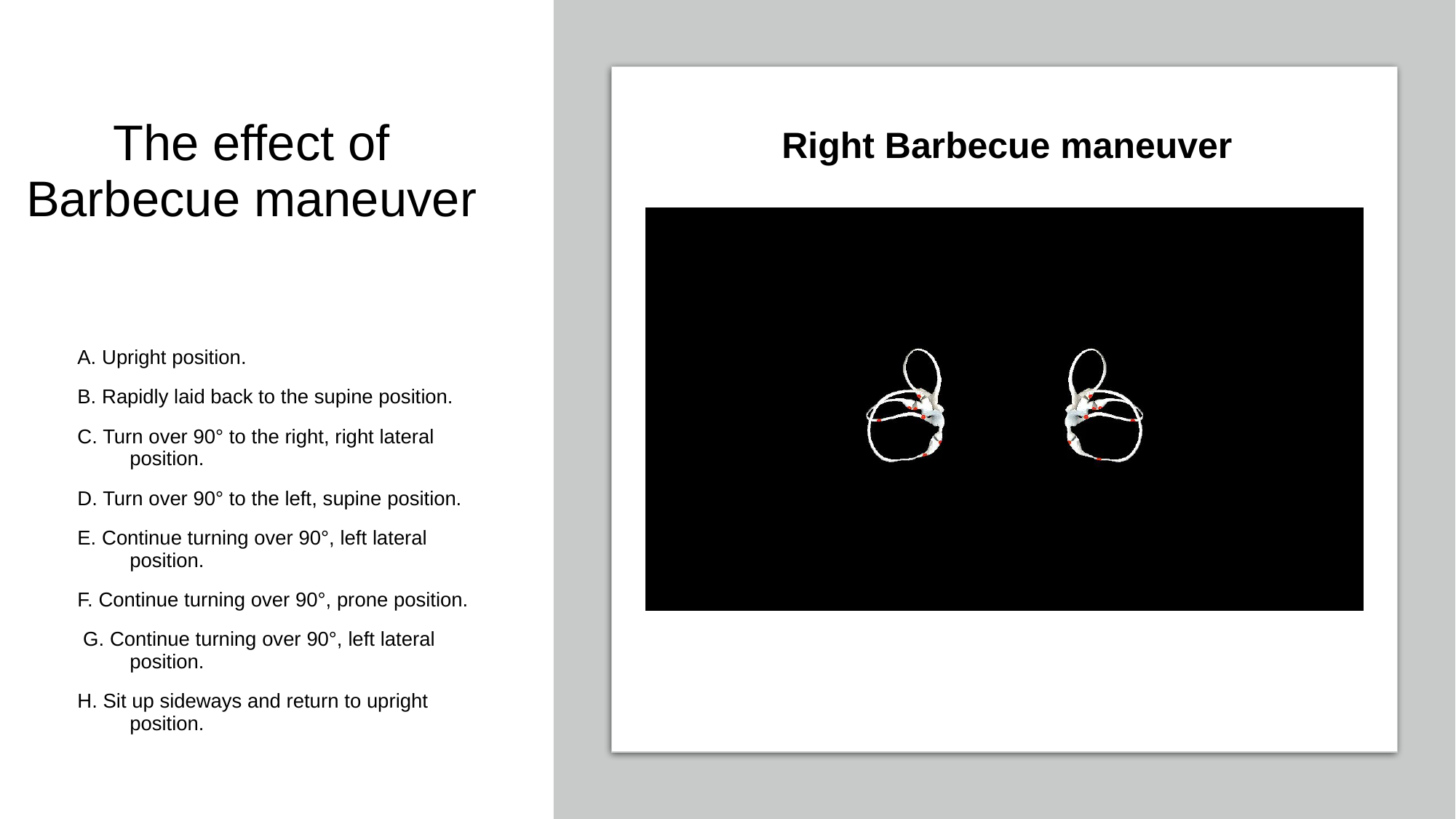

# The effect of Barbecue maneuver
Right Barbecue maneuver
A. Upright position.
B. Rapidly laid back to the supine position.
C. Turn over 90° to the right, right lateral position.
D. Turn over 90° to the left, supine position.
E. Continue turning over 90°, left lateral position.
F. Continue turning over 90°, prone position.
 G. Continue turning over 90°, left lateral position.
H. Sit up sideways and return to upright position.

## Slide 5
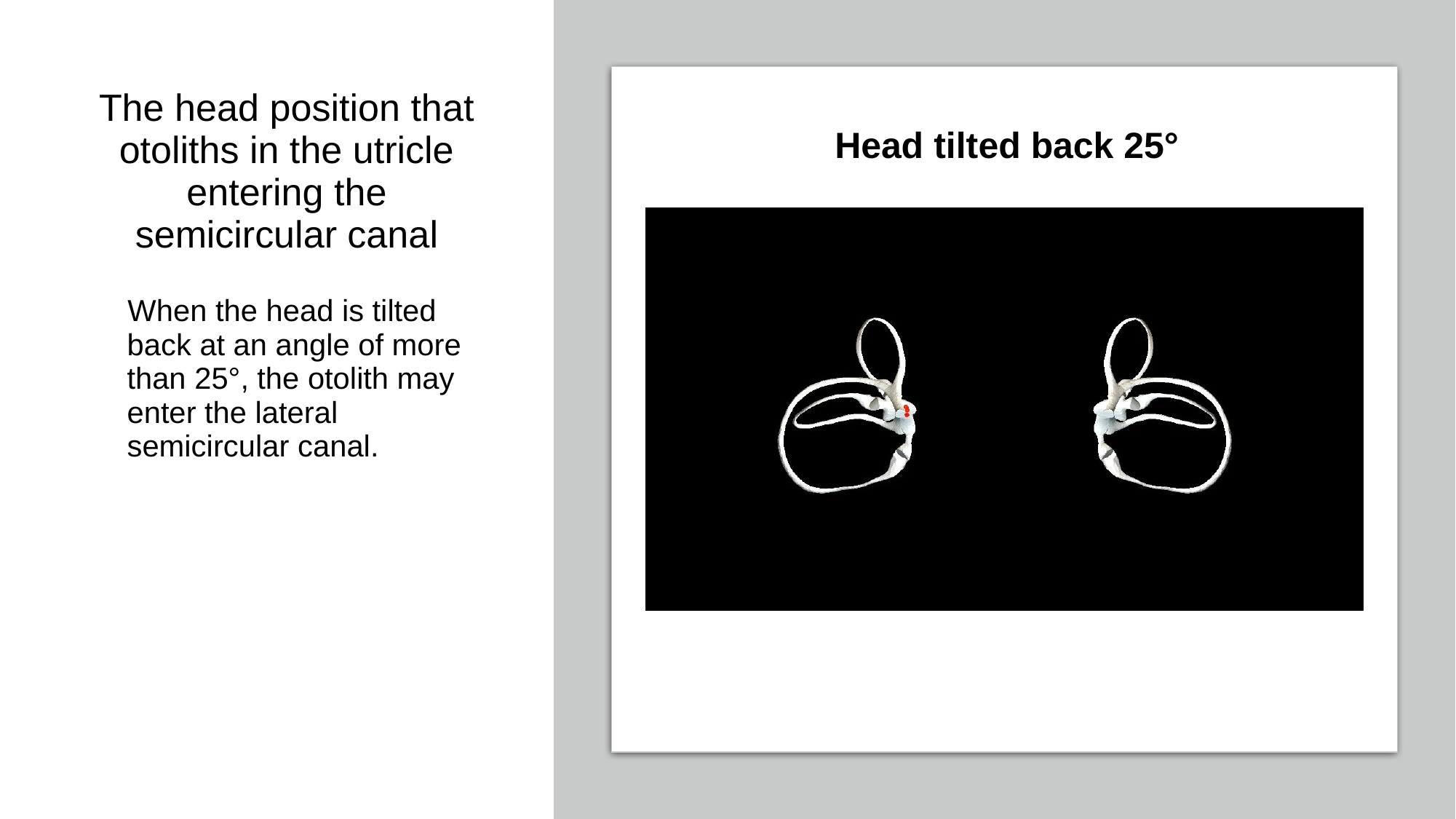

# The head position that otoliths in the utricle entering the semicircular canal
Head tilted back 25°
 When the head is tilted back at an angle of more than 25°, the otolith may enter the lateral semicircular canal.

## Slide 6
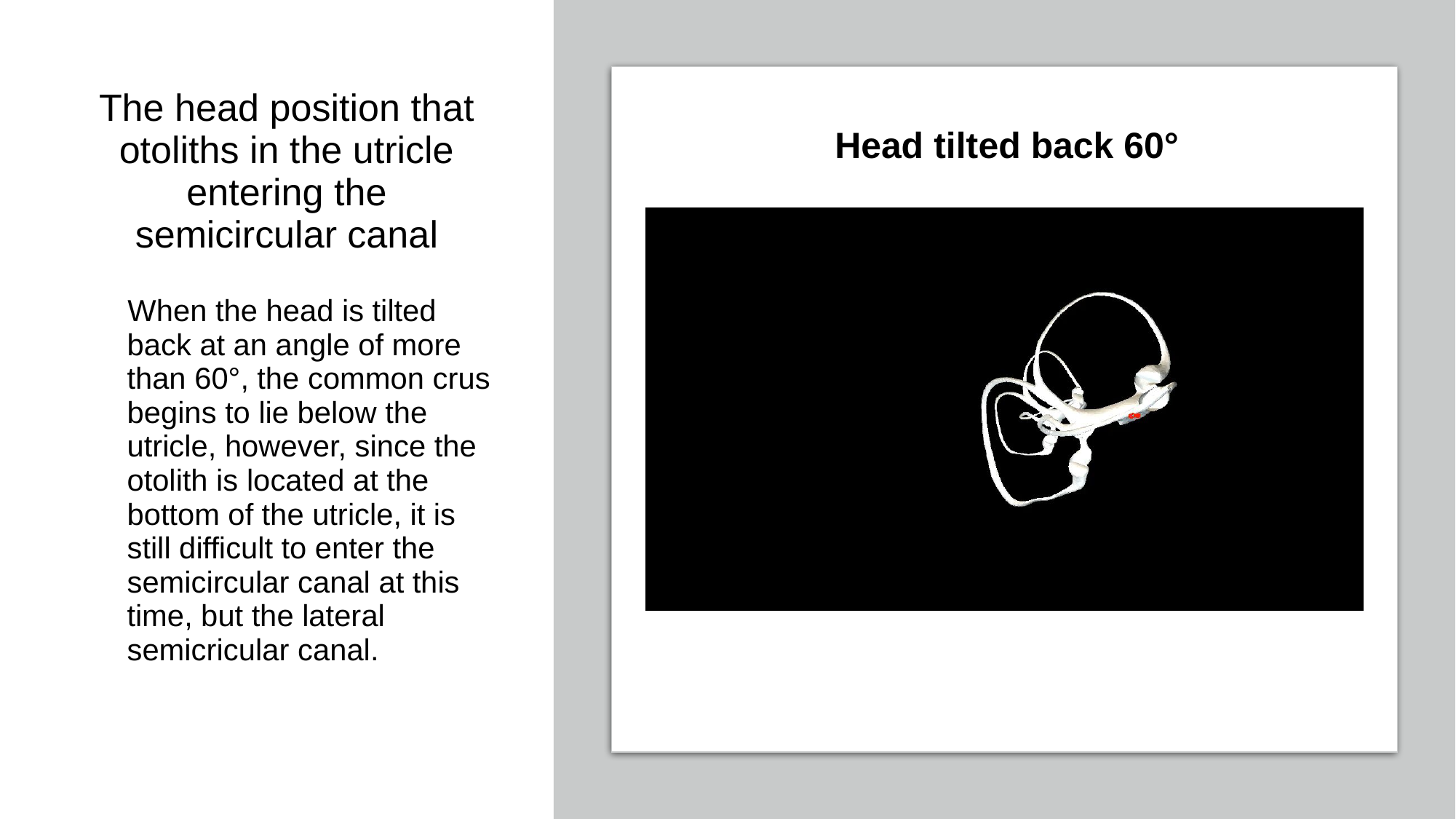

# The head position that otoliths in the utricle entering the semicircular canal
Head tilted back 60°
 When the head is tilted back at an angle of more than 60°, the common crus begins to lie below the utricle, however, since the otolith is located at the bottom of the utricle, it is still difficult to enter the semicircular canal at this time, but the lateral semicricular canal.

## Slide 7
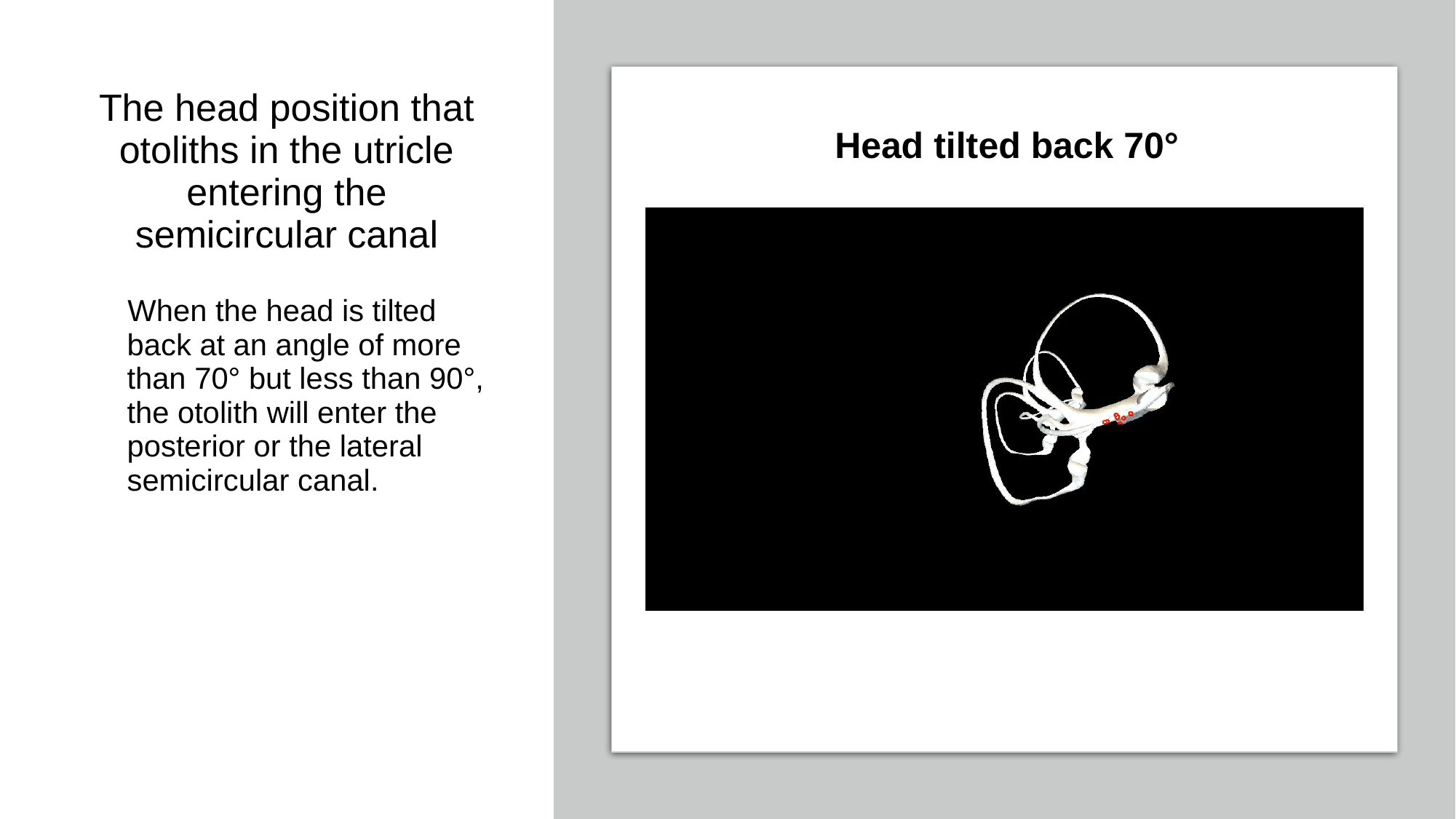

# The head position that otoliths in the utricle entering the semicircular canal
Head tilted back 70°
 When the head is tilted back at an angle of more than 70° but less than 90°, the otolith will enter the posterior or the lateral semicircular canal.

## Slide 8
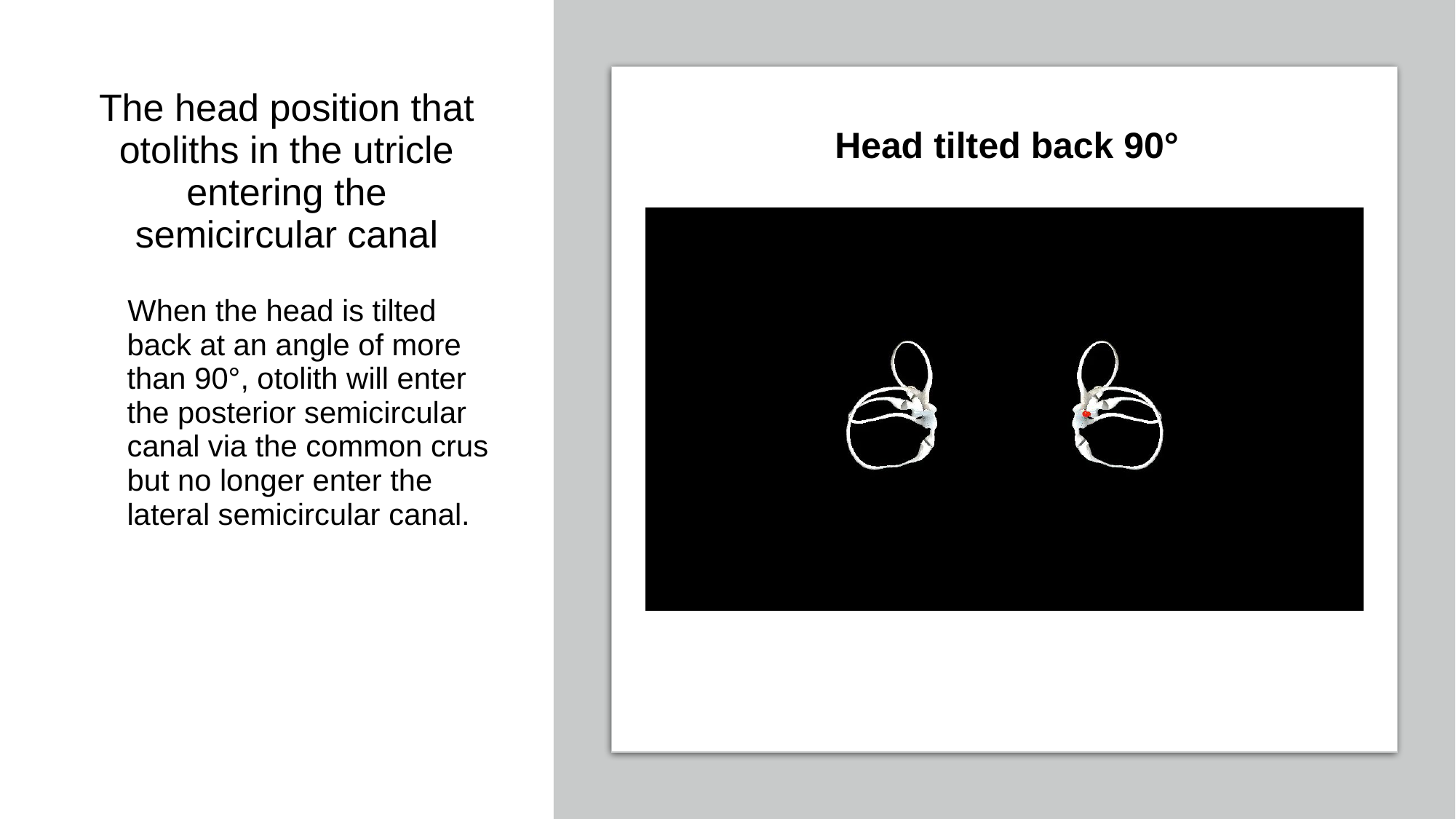

# The head position that otoliths in the utricle entering the semicircular canal
Head tilted back 90°
 When the head is tilted back at an angle of more than 90°, otolith will enter the posterior semicircular canal via the common crus but no longer enter the lateral semicircular canal.

## Slide 9
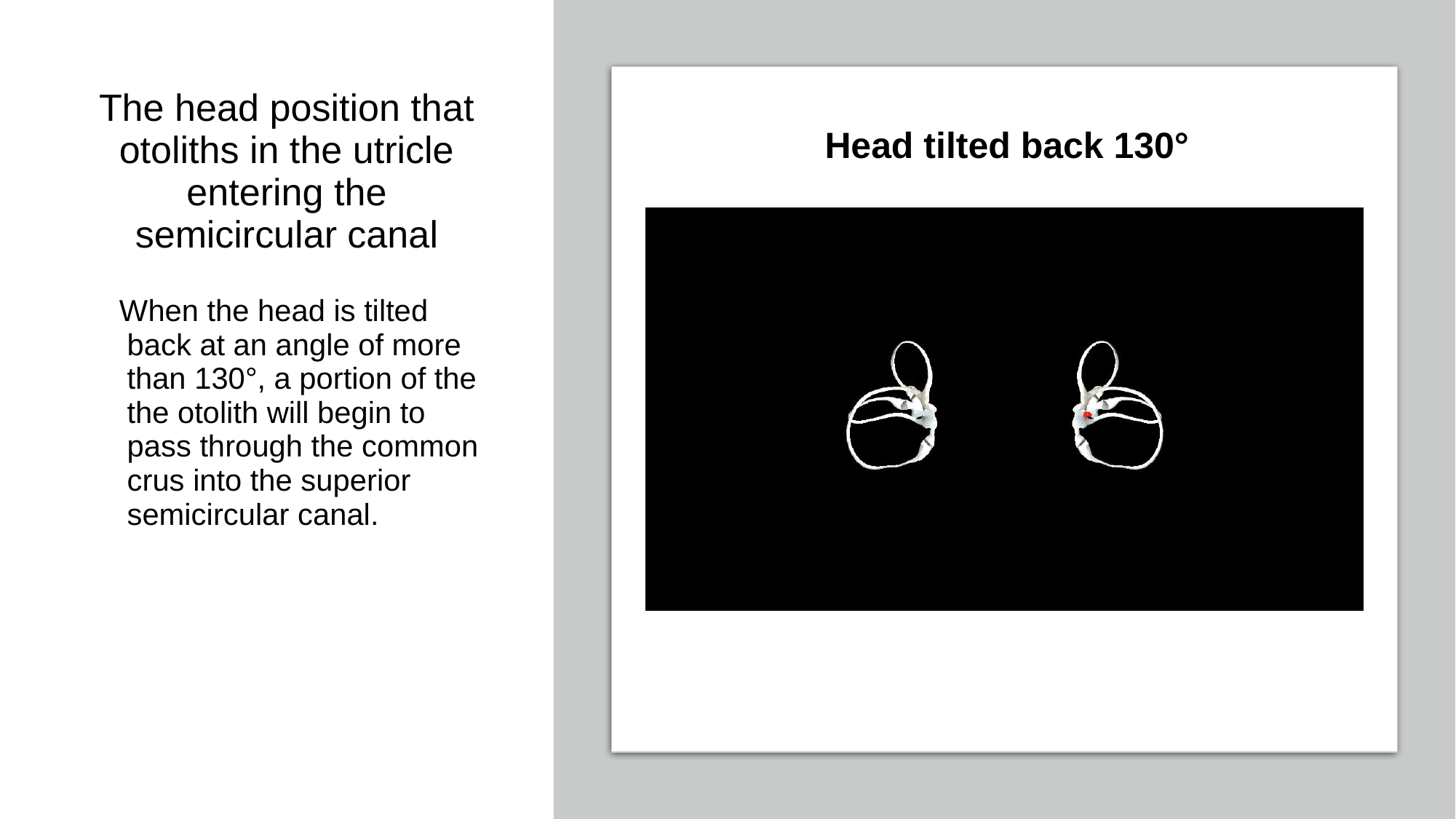

# The head position that otoliths in the utricle entering the semicircular canal
Head tilted back 130°
 When the head is tilted back at an angle of more than 130°, a portion of the the otolith will begin to pass through the common crus into the superior semicircular canal.

## Slide 10
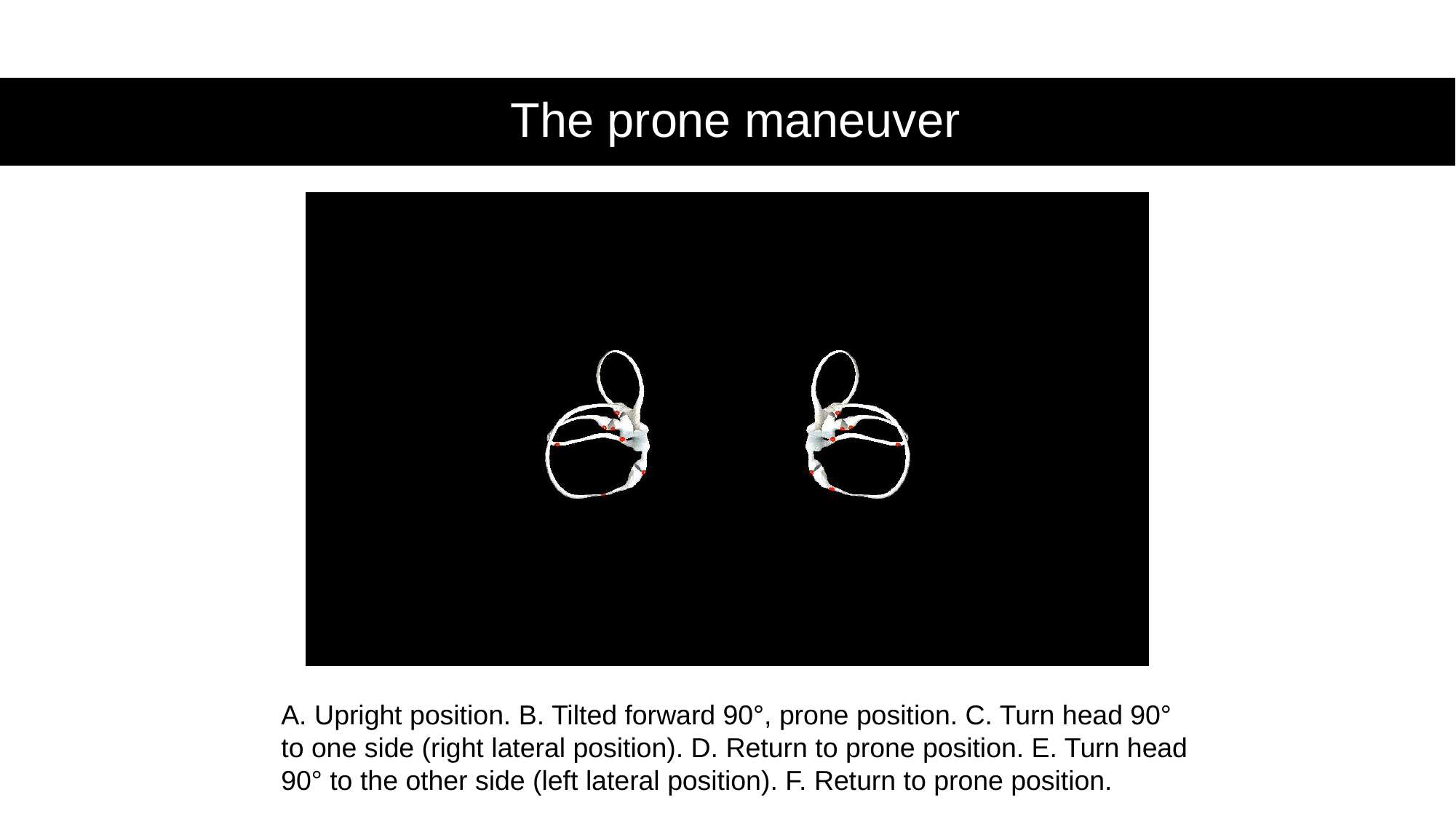

# The prone maneuver
A. Upright position. B. Tilted forward 90°, prone position. C. Turn head 90° to one side (right lateral position). D. Return to prone position. E. Turn head 90° to the other side (left lateral position). F. Return to prone position.
